# Vaccination with recombinant adenovirus expressing peste des petits ruminants virus-F or -H proteins elicits T cell responses to epitopes that arises during PPRV infection

**DOI:** 10.1186/s13567-017-0482-x

**Published:** 2017-11-21

**Authors:** José Manuel Rojas, Miguel Avia, Elena Pascual, Noemí Sevilla, Verónica Martín

**Affiliations:** Centro de Investigación en Sanidad Animal (CISA-INIA), Instituto Nacional de Investigación y Tecnología Agraria y Alimentaria, Valdeolmos, Madrid, Spain

## Abstract

Peste des petits ruminants virus (PPRV) causes an economically important disease that limits productivity in small domestic ruminants and often affects the livestock of the poorest populations in developing countries. Animals that survive PPRV develop strong cellular and humoral responses, which are probably necessary for protection. Vaccination should thus aim at mimicking these natural responses. Immunization strategies against this morbillivirus using recombinant adenoviruses expressing PPRV-F or -H proteins can protect PPRV-challenged animals and permit differentiation of infected from vaccinated animals. Little is known of the T cell repertoire these recombinant vaccines induce. In the present work, we identified several CD4^+^ and CD8^+^ T cell epitopes in sheep infected with PPRV. We also show that recombinant adenovirus vaccination induced T cell responses to the same epitopes, and led to memory T cell differentiation. T cells primed by these recombinant adenovirus vaccines expanded after PPRV challenge and probably contributed to protection. These data validate the use of recombinant adenovirus expressing PPRV genes as DIVA strategies to control this highly contagious disease.

**Electronic supplementary material:**

The online version of this article (10.1186/s13567-017-0482-x) contains supplementary material, which is available to authorized users.

## Introduction

Peste des petits ruminants virus (PPRV) causes an economically important disease that limits productivity in small domestic ruminants [1, 2]. Infection of naïve populations can be devastating, particularly in goats, leading to mortality rates of up to 90% [3–5]. PPRV is endemic in Central and East Africa, the Arabian Peninsula, Turkey, and India. Its prevalence in developing countries and its host spectrum often implies that the poorest populations within these countries are affected [6]. PPRV is highly contagious and in acute infections produces severe pyrexia, nasal and ocular discharges, pneumonia, enteritis and diarrhea [4, 5].

PPRV is a morbillivirus that belongs to the *Paramyxoviridae* family [7]. This genus of single-stranded negative sense enveloped RNA viruses causes relevant diseases (like measles or canine distemper) in human and animals. PPRV single-strand RNA genome encodes 6 structural and 2 non-structural proteins [1]. PPRV infection is immunosuppressive, which can lead to opportunistic pathogen infections that contribute to the high mortality and morbidity rates of infected animals [4, 8].

Current vaccines are based on live attenuated viruses that control the disease but cannot differentiate infected from vaccinated animals (the so-called DIVA approach) [9]. Traditional live attenuated vaccine can also produce immunosuppression, albeit to a lower extent than natural infections [10]. These drawbacks highlight the need for alternative vaccination strategies against this disease.

Most immunologically relevant determinants for protection in morbillivirus have been mapped to the surface fusion protein (F) and hemagglutinin (H) as well as to the nucleoprotein (NP) [11–15]. Recombinant vectors expressing these subunits thus represent attractive strategies for vaccination [16–22]. DIVA vaccines with recombinant adenovirus expressing the F or H protein can be protective in small ruminants [23–25], and potentially facilitate PPRV infection status monitoring.

Animals that survive PPRV infection develop a strong cellular and humoral response [11, 23, 26], which is probably essential for virus clearance and protection. In infection with the morbillivirus prototype measles virus (MeV), cellular and humoral immunity contribute to protection. Humoral immunity can protect against MeV re-infection, whereas cellular immunity controls virus clearance and dissemination [27–30]. Moreover, induction of neutralizing antibodies alone was also insufficient to protect cattle against rinderpest virus challenge, a virus closely related to PPRV [31]. It thus appears that protective natural immunity to morbilliviruses requires both humoral and cellular components of the adaptive immune system. Recombinant adenovirus vaccines should therefore aim at replicating the naturally occurring PPRV immunity. The immune responses that these vaccines elicit to the transgene are nonetheless not fully characterized. For instance determining whether the T cell repertoire they elicit is comparable to that of animals that recover from the disease could be indicative of vaccine efficacy.

In the present work, we set out to characterize T cell epitopes in mice and sheep from the main PPRV immunological determinants. We then assessed whether the responses to these immunogenic T cell epitopes overlapped in PPRV-infected and in recombinant adenovirus-vaccinated sheep. Finally, we measured the T cell functionality induced by these vaccines after heterologous PPRV challenge.

## Materials and methods

### Cells

Vero Dog-SLAM (VDS) and RMA/s cell lines were kindly provided by Dr Parida (IAH, Pirbright, UK) and Dr McArdle (The Nottingham Trent University, UK) respectively. HEK-293 (ATCC CRL-1573), VDS and RMA/s cell lines were maintained as described in [16, 32].

### PPRV and replication-defective recombinant adenovirus 5 (Ad5) vaccines

PPRV vaccine strain Nigeria 1975/1 (PPRV Nig’75; lineage II) and PPRV infective strain Ivory Coast 1989 (PPRV IC’89; lineage I) were kindly provided by Dr Batten (IAH, Pirbright, UK). PPRV stocks were grown in VDS cells, purified as described in [16], and inactivated as described in [33]. Replication-defective recombinant adenovirus 5 construction expressing the F (Ad5-F) or H (Ad5-H) gene from PPRV Nig’75 vaccine strain is described [16]. Recombinant adenovirus stocks were grown in HEK-293 cells and purified as described [23].

### PPRV peptide prediction and binding assays

Peptide binding to H-2^b^ haplotype from PPRV Nig’75-F (GenBank #CAJ01699.1), -H (GenBank #CAJ01700.1) and -NP (GenBank #CAA52454.1) proteins was predicted using three algorithms available on the web [34–37]. Peptide F10 was selected as a PPRV-F homologue to a cross-reactive morbillivirus T cell epitope [38, 39]. Peptides were synthesized by AltaBioscience (UK) and H-2D^b^ and H-2K^b^ binding assessed by flow cytometry using RMA/s cells and normalized to the lymphocytic choriomeningitic virus (LCMV) peptide gp (33–41) (KAVYNFATC) as described [32].

### Animal experimentation

Six week old female C57BL/6 mice (H-2^b^) were purchased from Harlan. Two-month old naïve female “Colmenareña” breed sheep were purchased from a certified provider. Experiments were performed in a disease-secure isolation facility (BSL3) at the Centro de Investigación en Sanidad Animal (CISA), in strict accordance with the recommendations of the Code for Methods and Welfare Considerations in Behavioural Research with Animals (Directive 86/609EC; RD1201/2005). Experiments were approved by the Committee on the Ethics of Animal Experiments (CEEA) of the Spanish Instituto Nacional de Investigación y Tecnología Agraría y Alimentaria (INIA) and the “Comisión de ética estatal de bienestar animal”. A 2-week acclimatization period prior to experimentation was observed during which animals were monitored daily for general health status.

### PPRV infection in mice and splenocyte preparation

Eight week-old C57BL/6 female were inoculated intraperitoneally with 1 × 10^5^ plaque forming units (PFU) PPRV IC’89 three times at 2 week interval. Mice were sacrificed 3 days after the last inoculation and splenocytes prepared and cultured as described [32].

### Sheep infection, peripheral blood mononuclear cell (PBMC) isolation and in vitro peptide restimulation

Sheep were randomly divided in 4 groups of 4 animals. PPRV IC’89 infection and recombinant adenovirus vaccination were performed as described in [23]. Control groups received two inoculations at 21 day interval of PBS or replication-defective recombinant empty Ad5 vaccine (Ad5-empty). Vaccinated groups received two immunizations at 21 day interval with Ad5-F or Ad5-H replication-defective recombinant vaccines [16]. All sheep were challenged intravenously with 1 × 10^6^ PFU heterologous virulent PPRV IC’89 strain on day 42. Clinical details of vaccination results are reported in [23]. PBMC were prepared [33] and stored frozen until use. For in vitro peptide expansion, PBMC were thawed, rested for 2 h, stimulated with 10 µg/mL peptide for 6–7 days, washed and then used in functional assays.

### ELISPOT and proliferation assays

Murine splenocytes or ovine PBMC (2 × 10^5^) were plated with 10 µg/mL peptide overnight. As positive control, cells were activated with 0.5 µg/mL concanavalin-A (Con-A) or 20 ng/mL phorbol myristyl acetate (PMA) + 1 µg/mL ionomycin. PPRV responses were measured with inactivated virus. Murine IFN-γ ELISPOT assays were performed according to the manufacturer’s protocol (Diaclone, France). Ovine IFN-γ ELISPOT assays are described in [33]. ELISPOT assays were considered valid when control well spot counts were below 25; and positive counts were > 10 and at least 2 standard deviations above background [23, 32, 40]. Proliferation assays were performed as described [33].

### Intracellular cytokine staining and flow cytometry

Ovine PBMC were stimulated overnight with 10 µg/mL PPRV peptide and brefeldin-A (5 µg/mL) added to the culture for the last 4 h incubation. As positive control, cells were stimulated with inactivated PPRV IC’89 or 20 ng/mL PMA + 1 µg/mL ionomycin. Cells were stained with anti-ovine CD45RO (ILA116A; KingFisher Biotech), anti-ovine CD4 (44.38; Biorad) and anti-ovine CD8 (38.65; Biorad), fixed in 4% paraformaldehyde, permeabilized with 0.2% saponin and stained with anti-bovine IFN-γ (CC302; Biorad). Appropriate isotype and fluorescence minus one-channel controls were used in these experiments for gate setting. Gating strategies are detailed in Additional file 1. FACScalibur was used for data acquisition and FlowJo software (Tree Star Inc.) for flow cytometry analysis.

### Flow cytometry cytotoxicity assays

For cytotoxicity assays, in vitro peptide-stimulated ovine PBMC were used as effector cells and autologous LPS-blast cells (differentiated with 2 µg/mL LPS and 7 µg/mL dextran sulfate [32, 41]) were used as target cells. LPS-blast cells were labelled with PKH67 green fluorescent linker [42], and pulsed with relevant peptide or inactivated PPRV IC’89. Effector cells (E) and targets cells (T) were incubated for 4 h at 37 °C in 96 U-bottom well plates at different ratios (E:T). Cells were then transferred to FACS tubes; dead cells labelled with propidium iodide (PI) (2 µg/mL); and samples immediately analyzed by flow cytometry. Gating strategy is given in Additional file 1. Target cells were gated on bright FL1^+^ cells. Positive maximum cell death controls (target cells in PBS + 0.2% saponin) and spontaneous cell death controls were used in all experiments. The percentage of specific target cell lysis was calculated following the formula: % specific lysis = 100 × (% PI^+^ target − % spontaneous death)/(% maximum death − % spontaneous death).

## Results

### Identification of PPRV T cell epitopes in sheep

**Figure 1:**
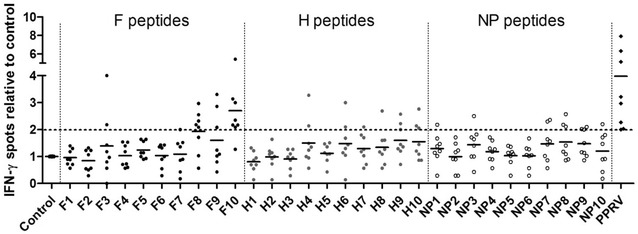
**PPRV epitope screening in infected sheep.** Sheep PBMC obtained 17–21 days post-PPRV (IC’89) infection were stimulated with predicted F, H or NP peptides and specific IFN-γ production assessed in ELISPOT assays. Data were normalized to spots detected in unstimulated cells. The dotted horizontal line represents the positive stimulation threshold (positive counts > 10 and at least 2 standard deviations above background; control well spot counts < 25).

**Figure 2:**
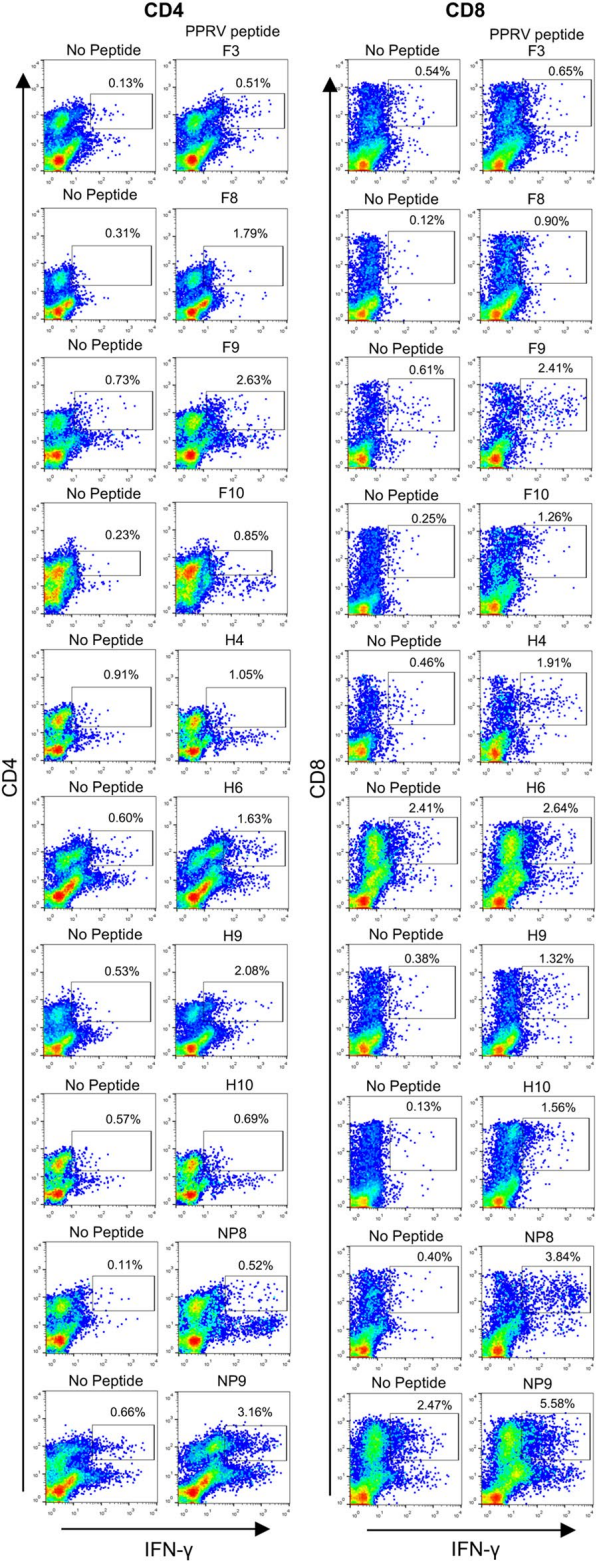
**IFN-γ CD4** ^**+**^ **and CD8** ^**+**^ **T cell responses to PPRV peptides.** Sheep PBMC obtained 17–21 days post-PPRV (IC’89) infection were expanded in vitro with peptide for 1 week and IFN-γ production was assessed by flow cytometry using intracellular staining. Representative dot-plots from 2 to 5 sheep are presented.

**Figure 3:**
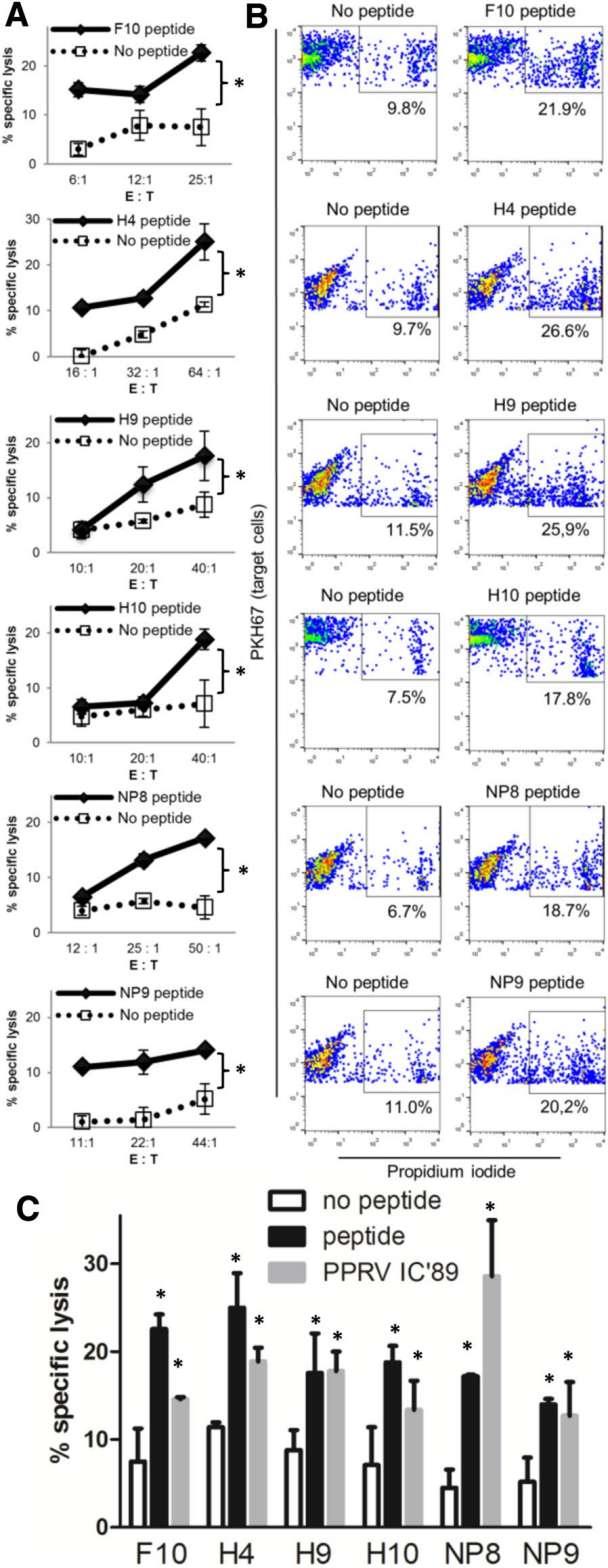
**Cytotoxic T lymphocyte responses to PPRV epitopes.** In vitro peptide-stimulated PBMC obtained 17–21 days post-infection from PPRV (IC’89)-infected sheep were used as effector cells (E) in cytotoxicity assays. Autologous LPS-blast cells were used as target cells (T) and labeled with PKH67 cell linker dye. Cell cytotoxicity was measured by propidium iodide staining at different effector cells (E) to target cells (T) ratios (E:T). Gating on bright PKH67^+^ and propidium iodide^+^ events by flow cytometry was used to calculate the percentage of target cell lysis. **A** Specific lysis (mean ± SD) to F10, H4, H9, H10, NP8 and NP9 peptides in PPRV IC’89 infected sheep. Student’s *t* test (peptide vs no peptide); *p < 0.05. **B** Representative dot-plots showing dead target cells gating. **C** Specific lysis (mean ± SD) of unpulsed, peptide-, or PPRV IC’89-pulsed autologous target cells. One way ANOVA with Dunnett’s post-test (peptide or PPRV IC’89 vs no peptide); *p < 0.05.

After characterizing PPPRV-F, -H and -NP binders to MHC class I molecules (Table 1) as well as their immunogenic potential in C57BL/6 mice (Additional file 2), the immunogenicity of these putative PPRV T cell epitopes was also explored in sheep. Peptide-specific IFN-γ production was measured in PBMC obtained 17–21 days after PPRV (IC’89) infection. Since previous work established that unvaccinated and Ad5-empty vaccinated sheep were naïve towards PPRV [23], sheep PBMC from both groups were used in these assays. As predicted, T cell responses to PPRV peptides depended on individual sheep as these animals are outbred. No differences in response frequency to peptides were observed between unvaccinated and Ad5-empty-vaccinated sheep and thus data are presented as one group. ELISPOT assays showed that peptides F3, F8, F9, F10, H4, H6, H9, H10, NP7, NP8 and NP9 induced significant IFN-γ production in at least 2 sheep (Figure 1). Since these peptides could contain epitopes that elicit T cell responses in several sheep, we focused our efforts on characterizing them further by intracellular IFN-γ staining (Figure 2). Peptides F8, F9, F10, H9, NP8 and NP9 induced IFN-γ production in CD4^+^ and CD8^+^ T cells. Peptides F3 and H6 induced specific IFN-γ production in CD4^+^ T cells, and peptides H4 and H10 induced IFN-γ production in CD8^+^ T cells. Peptide NP7 was not assessed in these assays due to limited PBMC availability. We also evaluated the cytotoxic T lymphocyte (CTL) response of CD8^+^ T cell epitopes against peptide-pulsed autologous target cells (Figure 3). Peptides F10, H4, H9, H10, NP8 and NP9 produced CTL responses (Figures 3A and B). Peptides F8 and F9 were also tested in these assays but no CTL activity was detected in the tested sheep (*n* = 2) (Table 2). F10, H4, H9, H10, NP8 and NP9 peptide-responding CTL could lyse target cells pulsed with inactivated PPRV IC’89 (Figure 3C). This indicates that these peptides are naturally processed and presented by target cells during PPRV infection.

**Table 1:** **PPRV-F, -H and -NP peptide prediction and binding assays to H-2D** ^**b**^ **and H-2** **K** ^**b**^ **molecules**

	PPRV protein (position)	Sequence	Predicted allele binding	Score SYFPEITHI	Score ProPred-I	Score NetMHC (predicted affinity nM)	Binding assay score^a^
F1	F(53–61)	KLMPNITAI	D^b^	23	138.312	365.28	++
F2	F(135–143)	QSLMNSQAI	D^b^	25	3281.040	34.37	++
F3	F(369–377)	GTTSNRFIL	D^b^	19	286.272	3413.74	+
F4	F(342–350)	NALYPMSPL	D^b^	16	30.240	1927.14	−
			K^b^	18	0.330	791.56	+++
F5	F(435–443)	SVYLHKIDL	K^b^	11	0.100	68.79	+
F6	F(278–286)	IAYPTLSEI	D^b^	18	4.234	3403.64	+
			K^b^	9	1.650	168.59	+
F7	F(7–21)	LVFLFLFPNTVTCQI	I-A^b^	NA	NA	79.2	NA
F8	F(117–131)	VALGVATAAQITAGV	I-A^b^	NA	NA	192.1	NA
F9	F(341–355)	QNALYPMSPLLQECF	I-A^b^	NA	NA	351.5	NA
F10	F(284–298)	LSEIKGVIVHKIEAI	Homologue to morbillivirus epitope [38, 39]	NA	NA	5014.3 (I-A^b^)	NA

H1	H(270–278)	FHMTNYLTV	D^b^	21	17.280	43.88	+++
H2	H(158–166)	AAVKSVEHI	D^b^	21	50.168	593.13	+++
H3	H(547–555)	RSSSYFYPV	K^b^	18	1.100	70.29	++
H4	H(549–557)	SSYFYPVRL	K^b^	18	5.500	8.75	+++
H5	H(551–559)	YFYPVRLNF	D^b^	7	0.000	42 098.88	+
			K^b^	7	0.120	1250.29	−
H6	H(44–52)	VMFLSLIGL	D^b^	15	14.334	6563.96	++
			K^b^	11	2.000	44.36	+++
H7	H(426–434)	VITSVFGPL	K^b^	21	12.000	96.10	+++
H8	H(441–455)	MDLYNNPFSRAAWLA	I-A^b^	NA	NA	42.2	NA
H9	H(427–441)	ITSVFGPLIPHLSGM	I-A^b^	NA	NA	74.8	NA
H10	H(448–462)	FSRAAWLAVPPYEQS	I-A^b^	NA	NA	319.6	NA

NP1	NP(29–37)	RGIKNVIIV	D^b^	19	38.880	265.97	+++
NP2	NP(72–80)	VMISMLSLF	D^b^	18	36.318	3827.32	−
			K^b^	11	0.030	97.12	+++
NP3	NP(294–302)	STIESLMNL	D^b^	18	12.773	7155.04	−
			K^b^	12	1.320	117.94	++
NP4	NP(335–343)	YAMGVGVEL	D^b^	16	30.240	6163.56	+
NP5	NP(354–362)	RSYFDPAYF	D^b^	11	0.091	4399.92	−
			K^b^	8	0.158	1703.38	−
NP6	NP(228–236)	SLRRFMVSL	K^b^	12	0.264	184.41	++
NP7	NP(298–306)	SLMNLYQQL	K^b^	22	26.400	85.56	+
NP8	NP(435–449)	REEVKAAIPNGSEGR	I-A^b^	NA	NA	95.2	NA
NP9	NP(327–341)	GAYPLLWSYAMGVGV	I-A^b^	NA	NA	290.8	NA
NP10	NP(176–190)	ILLAKAVTAPDTAAD	I-A^b^	NA	NA	355.6	NA

NA: not available, *+++* strong binder (ratio > 0.9), *++* moderate binder (0.7 < ratio < 0.9), *+* weak binder (0.5 < ratio < 0.7), ***−*** no binder (ratio < 0.5).

^a^Peptide score was ranked relative to LCMV gp33-41 peptide binding (ratio PPRV peptide MFI vs gp 33–41 MFI) [32].

**Table 2:** **Responses to F, H and NP peptides in PPRV IC’89-infected sheep**

Peptide	ELISPOT (positive/total sheep)	Intracellular IFN-γ staining	Cytotoxicity assay
F3	2/8	CD4^+^ (*n* = 2)	ND
F7	1/8	ND	ND
F8	5/8	CD4^+^/CD8^+^ (*n* = 4)	Not detected (*n* = 2)
F9	3/8	CD4^+^/CD8^+^ (*n* = 3)	Not detected (*n* = 2)
F10	7/8	CD4^+^/CD8^+^ (*n* = 5)	Positive (*n* = 2)
H4	2/8	CD8^+^ (*n* = 2)	Positive (*n* = 1)
H6	2/8	CD4^+^ (*n* = 2)	ND
H7	1/8	ND	ND
H8	1/8	ND	ND
H9	3/8	CD4^+^/CD8^+^ (*n* = 2)	Positive (*n* = 1)
H10	3/8	CD8^+^ (*n* = 2)	Positive (*n* = 1)
NP1	1/8	ND	ND
NP3	1/8	ND	ND
NP7	2/8	ND	ND
NP8	3/8	CD4^+^/CD8^+^ (*n* = 2)	Positive (*n* = 1)
NP9	3/8	CD4^+^/CD8^+^ (*n* = 2)	Positive (*n* = 2)
NP10	1/8	ND	ND

Due to limited PBMC numbers it was not possible to use all three techniques described on all sheep PBMC.

*n*: number of animal tested, ND: not done.

Our data thus show in sheep that peptides F3, F8, F9, F10, H6, H9, NP8 and NP9 contain CD4^+^ T cell PPRV epitopes and peptides F8, F9, F10, H4, H9, H10, NP8 and NP9 contain CD8^+^ T cell epitopes. Moreover, peptides F10, H4, H9, H10, NP8 and NP9 contain CTL epitopes. Based on the response frequency (Table 2), peptides F8, F9, F10, H9, H10, NP8 and NP9 are shared by several animals and could represent interesting targets for inclusion in PPRV vaccines.

### Recombinant Ad5 vaccination primes T cell responses to PPRV epitopes that arise during infection

**Figure 4:**
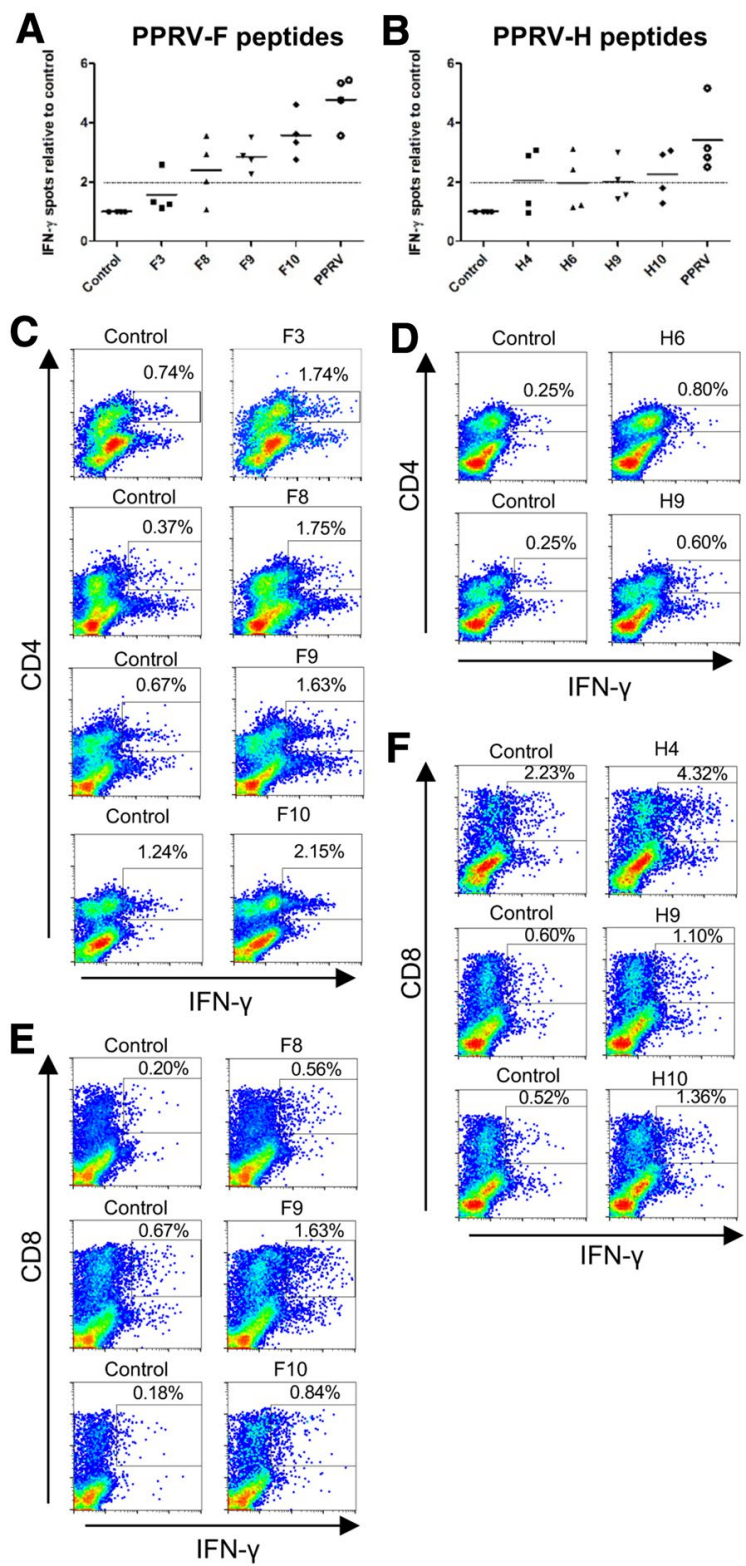
**Ad5-F and Ad5-H vaccination activates T cell responses to epitopes induced in PPRV infections.** The IFN-γ production to **A** F and **B** H epitopes was assessed in Ad5-F- and Ad5-H-vaccinated sheep, respectively by ELISPOT assays. The PBMC used in this experiment were from Ad5-F or Ad5-H immunized sheep obtained 21 days after booster vaccination and prior to PPRV challenge. Data are presented as mean IFN-γ spots normalized to control for each sheep. Inactivated PPRV IC’89 was used as positive control. The dotted horizontal line represents the positive stimulation threshold (positive counts > 10 and at least 2 standard deviations above background; control well spot counts < 25). **C**–**F** Intracellular IFN-γ staining and flow cytometry analysis used to identify T cell subsets responding to F and H epitopes in PBMC expanded with peptide for 1 week. Representative dot-plots of CD4^+^ T cell responses to **C** F epitopes and control (no peptide) in Ad5-F-vaccinated sheep and **D** H epitopes and control (no peptide) in Ad5-H-vaccinated sheep. Representative dot-plots of CD8^+^ T cell responses to **E** F epitopes and control (no peptide) in Ad5-F-vaccinated sheep and **F** H epitopes and control (no peptide) in Ad5-H-vaccinated sheep.

Since several T cell epitopes in PPRV-infected sheep were characterized, we wanted to determine whether vaccination with recombinant adenovirus expressing PPRV-F or -H proteins from the Nig’75 vaccine strain would trigger T cell responses to the F and H epitopes defined in animals infected with a heterologous strain (IC’89). In these experiments, we used PBMC from Ad5-F or Ad5-H immunized sheep obtained 21 days after booster vaccination and prior to PPRV challenge. PBMC from Ad5-F or Ad5-H-immunized sheep produced IFN-γ in response to peptides F3, F8, F9, F10 and H4, H6, H9 and H10 (Figures 4A and B), respectively. Vaccination therefore stimulated T cell responses towards F and H epitopes that coincide with some of those raised during PPRV infections. As observed in infected animals, peptides F3, F8, F9, F10, H6 and H9 induced CD4^+^ T cell IFN-γ production (Figures 4C and D) while peptides F8, F9, F10, H4, H9 and H10 induced CD8^+^ T cell IFN-γ production (Figures 4E and F) in recombinant Ad5-vaccinated animals. Thus Ad5-F or Ad5-H vaccination in sheep induced CD4^+^ and CD8^+^ T cell responses that are directed to several epitopes presented also during the course of a natural PPRV IC’89 infection.

### Recombinant Ad5 vaccines produce memory T cells that expand after PPRV challenge

**Figure 5:**
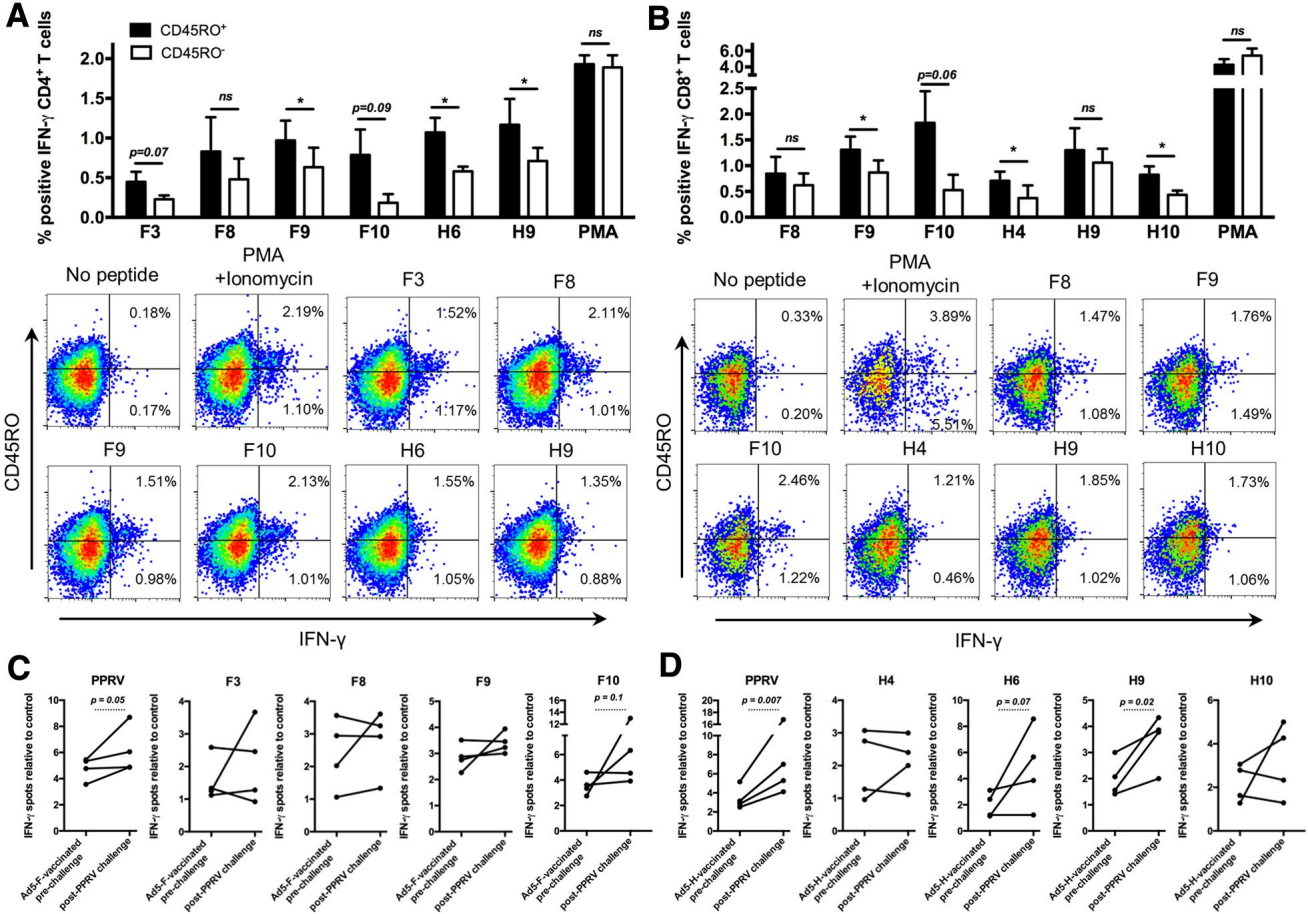
**Ad5-F and Ad5-H vaccination produces functional memory T cells to PPRV immunogenic peptides.** PBMC from Ad5-F- or Ad5-H-vaccinated sheep (21 days after booster and prior to PPRV challenge) were stimulated with PPRV immunogenic peptides and stained for CD4, CD8, the memory marker CD45RO and intracellular IFN-γ. **A** Mean (± SEM) IFN-γ production in CD45RO^+^ and CD45RO^−^ **A** CD4^+^ or **B** CD8^+^ T cells in two to four Ad5-F- or Ad5-H-immunized sheep is plotted. PMA and ionomycin was used as positive control. Representative IFN-γ and CD45RO stainings in **A** CD4^+^ or **B** CD8^+^ T cells from Ad5-F- or Ad5-H-vaccinated sheep in response to PPRV immunogenic peptide stimulation. Student’s *t* test (CD45RO^+^ vs CD45RO^−^); *p < 0.05. IFN-γ production in PBMC from **C** Ad5-F- or **D** Ad5-H-vaccinated sheep was measured pre-challenge and after PPRV IC’89 challenge by ELISPOT assays. Data were normalized to spots detected in unstimulated cells. *p* ≤ 0.1 in paired Student’s *t* tests are shown.

We next evaluated whether Ad5-F or Ad5-H vaccination produced memory T cells by measuring the expression of the memory marker CD45RO [43, 44] on IFN-γ-producing cells that responded to PPRV immunogenic peptides. Anti-PPRV CD4^+^ (Figure 5A) and CD8^+^ (Figure 5B) IFN-γ-producing T cells predominantly expressed the memory marker CD45RO, which indicated that these cells are antigen-experienced. Ad5-F and Ad5-H vaccination therefore led to anti-PPRV memory T cell differentiation.

To evaluate anti-PPRV memory T cell function, we measured the amplitude of the T cell responses by ELISPOT assays in Ad5-F- or Ad5-H-vaccinated sheep prior to PPRV challenge (21 days after booster vaccination) and 15 days after challenge. PPRV (IC’89) challenge increased T cell responses to the virus and to several F (Figure 5C) and H (Figure 5D) immunogenic peptides. These data confirm that Ad5-F and Ad5-H can induce a productive memory T cell response that can be reactivated after PPRV challenge.

## Discussion

Recombinant adenovirus vector vaccines are highly immunogenic and induce innate and adaptive immunity. Adenoviruses are recognized through pattern recognition receptors in transduced cells [45–48] thus producing adjuvancy towards the transgene [49]. Adenoviruses induce B, CD4^+^ T, CD8^+^ T cell responses to the adenovirus [50–54] and to the inserted transgene [16, 17, 22–24, 55–57]. Recombinant Ad5 immunization therefore represents a promising tool for recombinant vaccine development.

Recombinant adenovirus expressing the protein F or H from the economically important morbillivirus PPRV are immunogenic [16, 17, 22], and can protect sheep and goats from virulent virus challenge [23–25]. These recombinant adenovirus vaccines can also overcome PPRV-induced immunosuppression [23], an aspect of PPRV infection that can result in infected animals succumbing to opportunistic infections. Adenoviral vectors are known to induce strong T cell responses to the transgene. However, the extent to which this T cell response mimics the protective repertoire induced by the pathogen is not fully understood. In the present work we detect a clear overlap between the CD4^+^ and CD8^+^ T cell responses triggered after recombinant Ad5 vaccination and PPRV experimental infections. Responses to the tested PPRV-F and -H epitopes elicited during PPRV infections were also induced by Ad5 vaccination.

CD8^+^ T cell responses to transgene expressed by adenovirus depend on dose and route of injection; with high antigen dose leading to CD8^+^ T cell activation but impaired memory development [55]. This impaired memory differentiation in CD8^+^ T cells after high dose adenoviral vaccine inoculations could be due to antigen persistence that exhausts CD8^+^ T cells, in a similar manner to that observed in LCMV chronic infections [58, 59]. An inverse correlation between vector dose and T cell response to the transgene has been observed in adenoviral vaccinations [60]. Repeated adenovirus vaccine administrations at moderate doses however do not affect CD8^+^ T cell memory development [61]. In the present work, sheep were immunized twice with 10^8^ PFU of Ad5-F or Ad5-H vaccines, a low dose compared to murine T cell exhaustion studies that often used more than 10^9^ PFU. This vaccine dose in sheep permitted CTL generation and memory differentiation of CD8^+^ T cells specific for PPRV-F and -H epitopes. Since CD8^+^ T cells limit the dissemination of the morbillivirus prototype MeV [27–30], the activation of these memory anti-PPRV CD8^+^ T cells could have thus contributed to sheep protection against virulent virus challenge [23]. Another study found that a single inoculation at similar concentrations of recombinant adenovirus vaccine expressing PPRV-F or -H could protect goats when challenged 12 weeks post-immunization with infectious PPRV [25]. This implies that even a moderate single dose of Ad5-F or Ad5-H is likely to induce memory T cell differentiation.

Recombinant adenovirus vaccines can also be manipulated to alter epitope hierarchy and favor the CD8^+^ T cell response to subdominant epitopes [62]. This strategy could be used in sheep to broaden the T cell repertoire in viral infections that narrowly focus the T cell response [63, 64]. Importantly, adenovirus vaccines can also produce inflationary CD8^+^ T cell memory to some epitopes [65–67]. This memory population is characterized by the enrichment in peripheral organs of functional antigen-specific CD8^+^ T cells at high frequency. These cells could thus constitute a first line of peripheral defense capable of swiftly responding to viral infections. We observed increased T cell responses to some PPRV-F and -H peptides after virus challenge, which could indicate that Ad5-F and Ad5-H vaccination induced inflationary memory responses to some PPRV epitopes, although further characterization of the responding T cells is necessary to confirm this observation.

Adenovirus vaccines also produce mucosal trafficking of antigen-specific CD4^+^ T cells [68–70]. In the present study, we detected anti-PPRV memory CD4^+^ T cells in PBMC after recombinant Ad5 vaccination. It would be interesting to determine in future work whether these antigen-specific experienced cells are detected in the mucosa of Ad5-F- or Ad5-H-vaccinated sheep, particularly in the oral mucosa that PPRV uses as a gateway for infection. Recombinant adenovirus vaccination has therefore the potential to produce inflationary CD8^+^ T cell responses to the transgene and recruit CD4^+^ T cells to the mucosa, both of which could contribute to Ad5-F and Ad5-H vaccine efficacy.

Overall, we identified several PPRV-F, -H and -NP T cell epitopes after PPRV infection in sheep and mice (Additional file 2). The immunogenic regions identified in the present work could be useful for monitoring T cell responses induced by novel recombinant vaccines, which potential efficacy is usually tested in murine models. This is particularly relevant in morbillivirus vaccines, in which induction of a strong T cell response is probably necessary for successful vaccination. Sheep immunization with recombinant Ad5 vaccines expressing PPRV-F or -H genes mimicked the analyzed T cell repertoire induced by PPRV infection. Ad5-F and Ad5-H vaccination induced CD4^+^ and CD8^+^ T cell memory differentiation that could be re-activated by virulent PPRV challenge. These anti-PPRV memory T cells probably contributed to Ad5-F and Ad5-H protective effects. These data validate the use of recombinant Ad5 vaccines for PPRV control. A better understanding of the T cell memory response induced by recombinant adenovirus vaccines could ultimately improve memory cell activation by these therapies.

## Additional files

**Additional file 1.** **Gating strategies for IFN-γ detection, cytotoxicity assays and CD45RO expression.** (A) For IFN-γ detection, cells were selected by FSC/SSC discrimination. Gating for IFN-γ^+^ events was set using fluorescence minus one antibody (isotype) staining for CD4^+^ and CD8^+^ events. This gating was then maintained to measured IFN-γ^+^ events in stimulated cells. (B) In cytotoxicity assays, FSC/SSC discrimination was applied to gate putative live and dead cell events. Target cells labelled with the cell membrane marker PKH67 were first run on the cytometer to set up the target cell gate (PKH67^+^ events). Propidium iodide was used to discriminate live and dead cells. Bright PKH67^+^ and propidium iodide^+^ events were considered dead target cells. For each target cells, spontaneous and maximum cell death controls were acquired. In cytotoxicity co-culture assays, specific target cell lysis was assessed in the bright PKH67^+^gate. (C) For CD45RO expression, cells were first selected selected by FSC/SSC discrimination followed by CD4 or CD8 gating. Within these CD4^+^ or CD8^+^ gates, CD45RO^+^ gate was set using fluorescence minus one antibody (isotype) staining.

**Additional file 2.** **PPRV T cell repertoire in mice: identification of immunoreactive PPRV-T cell epitopes in H-2** ^**b**^ **context.** To determine whether recombinant adenovirus vaccination elicits T cell responses to determinants that are also targeted during PPRV infection, we first set out to identify T cell epitopes in mice. Since few PPRV T cell epitopes have been reported [11–14], we attempted to describe new determinants in our experimental settings. We focused our approach on the F, H and NP proteins as T cell determinants involved in morbillivirus responses are usually mapped to these. Peptides predicted to bind to murine H-2^b^ molecules (D^b^, K^b^ or I-A^b^) were selected using algorithms available online (Table 1) [34–37] and synthesized. Using the TAP-deficient cell line RMA/s, we performed binding assays for MHC class I predicted binders. Most peptides bound their predicted MHC class I molecules. Only peptide NP5 did not bind to D^b^ or K^b^ molecules. All 3 algorithms employed predicted D^b^ binders quite accurately. The NetMHC prediction was nonetheless more accurate for K^b^ binding than ProPred-I or SYFPEITHI. PPRV-F, -H and -NP peptide immunogenicity data in C57BL/6 mice are presented in the figure of Additional file 2. PPRV peptide immunogenicity was tested on splenocytes from C57BL/6 PPRV-infected mice (IC’89; 1 × 10^6^ PFU) using (A–C) IFN-γ ELISPOT and (D–F) proliferation assays. Responses to predicted peptides from PPRV (A and D) -F, (B and E) -H and (C and F) -NP proteins were measured in 8 mice per group. ELISPOT data are presented as average spots counted for 2 × 10^5^ cells and proliferation as stimulation index (cpm ratio in test vs control). One-way ANOVA (Dunnett’s post-test: peptides vs control); *p < 0.05; **p < 0.01; ***p < 0.001. (A–C) Significant IFN-γ production was detected to peptides F2, F3, F7, F8, F9, F10, H2, H5, H6, H9, NP5, NP8, NP9 and NP10. (D–F) Significant splenocyte proliferation was detected to peptides F2, F7, H2, H5 and H9. Peptides F9 and F10 only tended to induce higher proliferation.
