# Supplementary figures and images for: Vaccination with recombinant adenovirus expressing peste des petits ruminants virus-F or -H proteins elicits T cell responses to epitopes that arises during PPRV infection
Source: Vet Res. 2017 Nov 21;48:79. doi: 10.1186/s13567-017-0482-x (PMC5697415; doi:10.1186/s13567-017-0482-x)

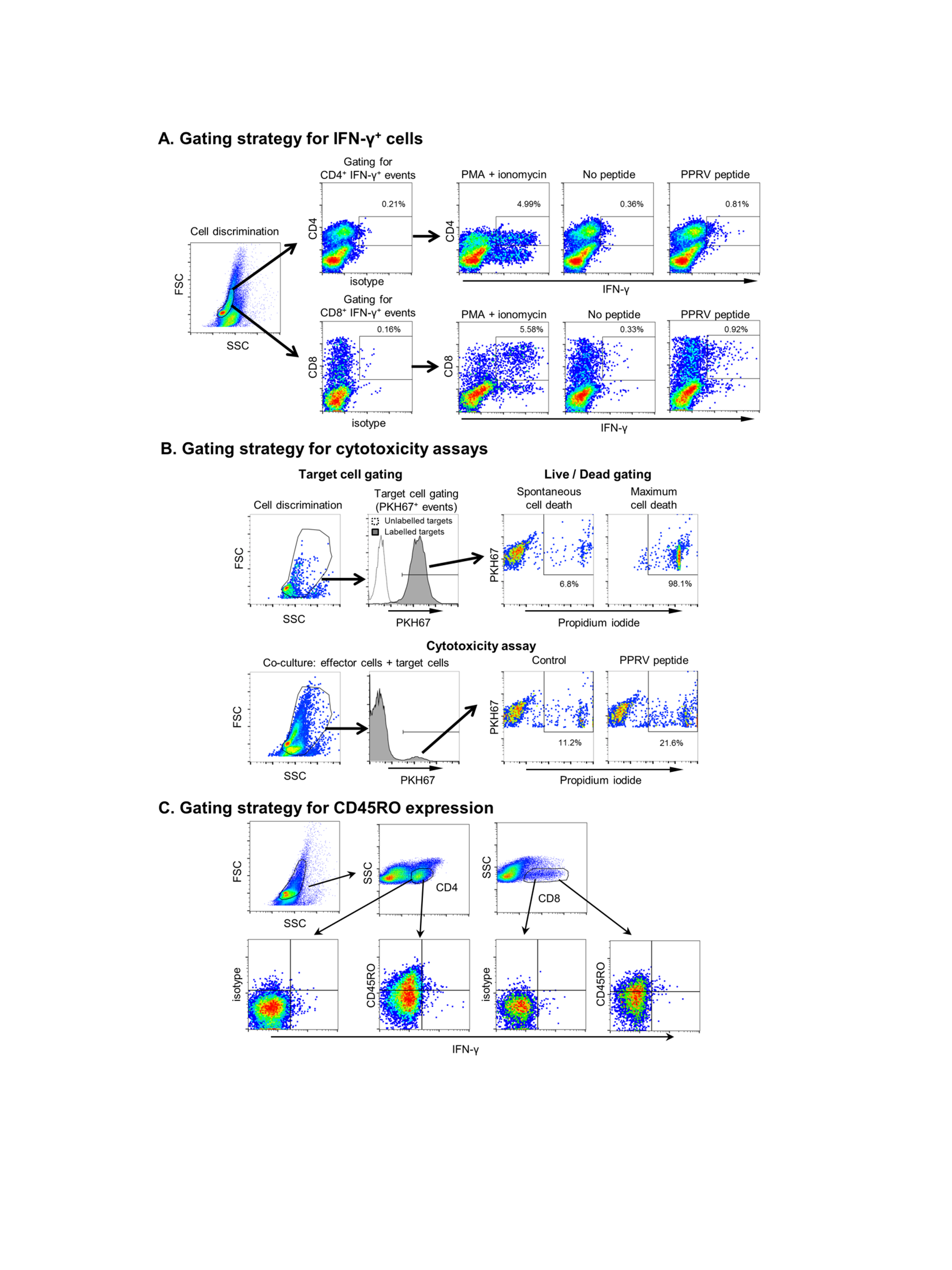

Supplement: Supplementary file 1 — Additional file 1. Gating strategies for IFN-γ detection, cytotoxicity assays and CD45RO expression. (A) For IFN-γ detection, cells were selected by FSC/SSC discrimination. Gating for IFN-γ+ events was set using fluorescence minus one antibody (isotype) staining for CD4+ and CD8+ events. This gating was then maintained to measured IFN-γ+ events in stimulated cells. (B) In cytotoxicity assays, FSC/SSC discrimination was applied to gate putative live and dead cell events. Target cells labelled with the cell membrane marker PKH67 were first run on the cytometer to set up the target cell gate (PKH67+ events). Propidium iodide was used to discriminate live and dead cells. Bright PKH67+ and propidium iodide+ events were considered dead target cells. For each target cells, spontaneous and maximum cell death controls were acquired. In cytotoxicity co-culture assays, specific target cell lysis was assessed in the bright PKH67+gate. (C) For CD45RO expression, cells were first selected selected by FSC/SSC discrimination followed by CD4 or CD8 gating. Within these CD4+ or CD8+ gates, CD45RO+ gate was set using fluorescence minus one antibody (isotype) staining. [file 13567_2017_482_MOESM1_ESM.tif]

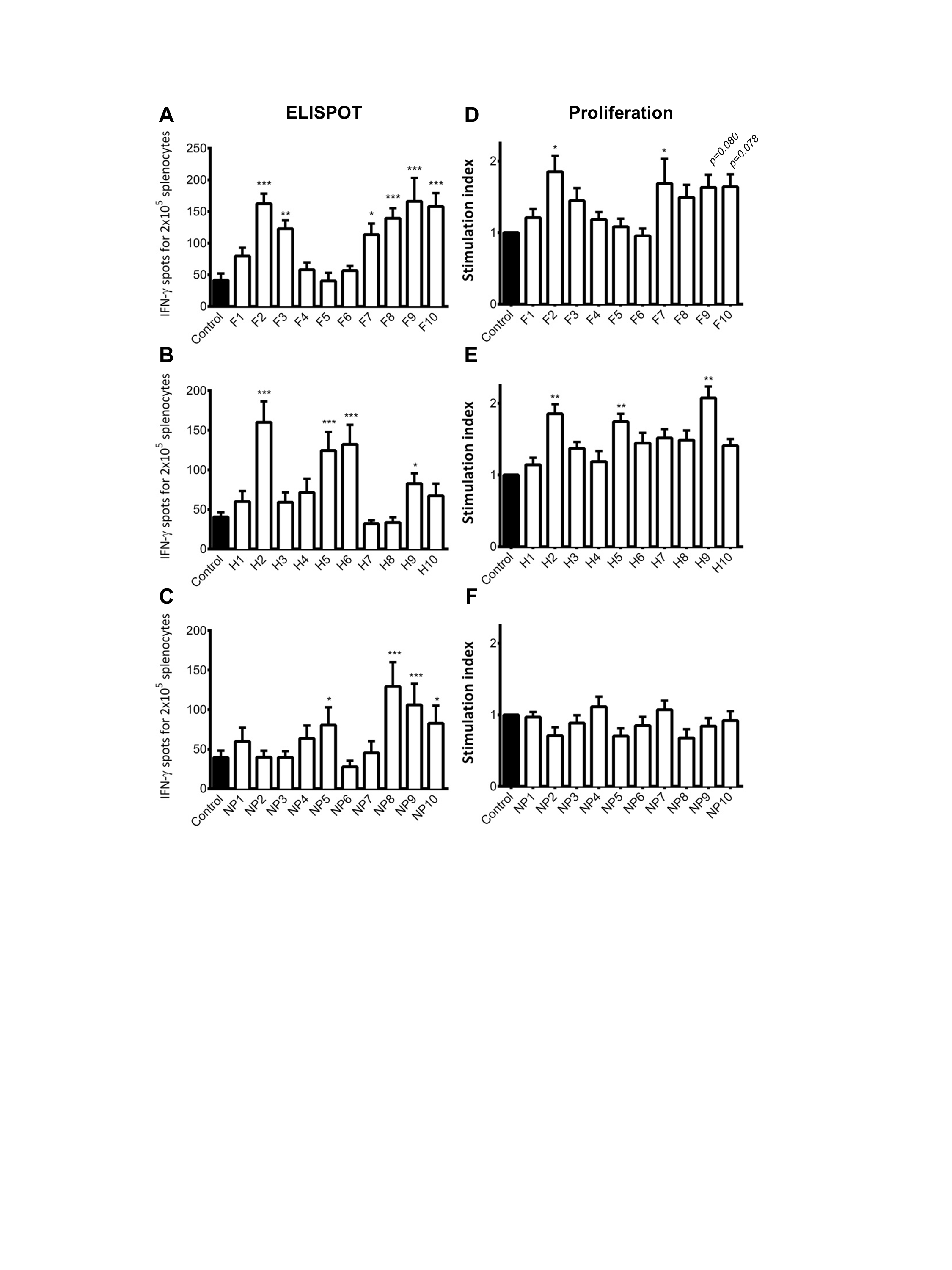

Supplement: Supplementary file 2 — Additional file 2. PPRV T cell repertoire in mice: identification of immunoreactive PPRV-T cell epitopes in H-2 b context. To determine whether recombinant adenovirus vaccination elicits T cell responses to determinants that are also targeted during PPRV infection, we first set out to identify T cell epitopes in mice. Since few PPRV T cell epitopes have been reported [11–14], we attempted to describe new determinants in our experimental settings. We focused our approach on the F, H and NP proteins as T cell determinants involved in morbillivirus responses are usually mapped to these. Peptides predicted to bind to murine H-2b molecules (Db, Kb or I-Ab) were selected using algorithms available online (Table 1) [34–37] and synthesized. Using the TAP-deficient cell line RMA/s, we performed binding assays for MHC class I predicted binders. Most peptides bound their predicted MHC class I molecules. Only peptide NP5 did not bind to Db or Kb molecules. All 3 algorithms employed predicted Db binders quite accurately. The NetMHC prediction was nonetheless more accurate for Kb binding than ProPred-I or SYFPEITHI. PPRV-F, -H and -NP peptide immunogenicity data in C57BL/6 mice are presented in the figure of Additional file 2. PPRV peptide immunogenicity was tested on splenocytes from C57BL/6 PPRV-infected mice (IC’89; 1 × 10^6^ PFU) using (A–C) IFN-γ ELISPOT and (D–F) proliferation assays. Responses to predicted peptides from PPRV (A and D) -F, (B and E) -H and (C and F) -NP proteins were measured in 8 mice per group. ELISPOT data are presented as average spots counted for 2 × 10^5^ cells and proliferation as stimulation index (cpm ratio in test vs control). One-way ANOVA (Dunnett’s post-test: peptides vs control); *p < 0.05; **p < 0.01; ***p < 0.001. (A–C) Significant IFN-γ production was detected to peptides F2, F3, F7, F8, F9, F10, H2, H5, H6, H9, NP5, NP8, NP9 and NP10. (D–F) Significant splenocyte proliferation was detected to peptides F2, F7, H2, H5 and H9. Peptides F9 and F10 [file 13567_2017_482_MOESM2_ESM.tif]
